# Comparison in safety of chemotherapy protocols for blood cancers: toxicity of H-CVAD versus GELA/BURKIMAB/PETHEMA LAL

**DOI:** 10.3332/ecancer.2021.1206

**Published:** 2021-03-22

**Authors:** Alberto Marín-Sánchez, Gonzalo Martínez-Fernández, Irene Gómez-Catalán, Mari Carmen Montoya-Morcillo, Jesús Lorenzo Algarra, Ángela Ibañez García, Francisco Hernández-Fernández, Juan Ramón Romero-Macías

**Affiliations:** 1 Haematology Department, Complejo Hospitalario Universitario de Albacete, Calle Hermanos Falcó 37, 02008 Albacete, Spain; 2 Nephrology Department, Hospital Universitario Reina Sofía, Murcia, Spain; 3 Neurology Department, Complejo Hospitalario Universitario de Albacete, Calle Hermanos Falcó 37, 02008 Albacete, Spain

**Keywords:** blood cancers, toxicity, infections

## Abstract

**Background and objective:**

The Hyper-CVAD/Methotrexate-Cytarabine (H-CVAD/MTX-AraC) chemotherapy protocol has been one of the standard treatments for blood cancers, such as Mantle cell lymphoma (MCL), Burkitt’s lymphoma (BL) and B-cell and T-cell acute lymphoblastic leukaemia (ALL). Due to high toxicity, it has been progressively replaced with new specific regimens with a better safety profile (GELA protocol for MCL, BURKIMAB for BL and PETHEMA for B-cell and T-cell ALL). The objective of this study is to analyse the toxicity and infectious complications of these therapeutic regimens, as well as the event free survival (EFS).

**Patients and methods:**

This is a retrospective and descriptive observational study of 81 patients, comparing 42 patients treated with H-CVAD/MTX-AraC (group A) versus 39 patients treated with GELA/BURKIMAB/PETHEMA (group B).

**Results:**

All patients in group A developed pancytopenia, but in group B 74.4% neutropenia, 51.3% thrombocytopenia and 69.2% anaemia. The total number of infections in group A was higher than in group B: 154 versus 48, 3.67 versus 1.23 per patient and 0.59 versus 0.25 per cycle. Likewise, febrile neutropenia happened: 106 versus 21 cases, 2.52 versus 0.52 per patient and 0.41 versus 0.11 per cycle. EVS is higher in group B: 33% versus 79% (2-year), and 24% versus 69% (5-year).

**Conclusions:**

Current therapeutic protocols have shown higher EFS due to better safety profile, with less haematological, neurological and haemorrhagic toxicity, as well as lower rates of infectious complications.

## Introduction

Lymphomas and acute leukaemias are blood cancers with high morbidity and mortality rates. In order to cure them, intensive anticancer therapy is required [1] However, this can lead to significant toxicity and systemic complications. One of the most undesirable effects of the treatment regimens is haematological toxicity, specifically neutropenia, as it predisposes patients to the risk of infection by different germs [2].

Chemotherapy treatment targeting different types of haematological malignancies has evolved substantially in recent years [3]. Since 2000, the combined Hyper-CVAD/Methotrexate-Cytarabine (H-CVAD/MTX-AraC) protocol has been the treatment regimen of choice used in many centres for cancers such as Mantle cell lymphoma (MCL) [4], Burkitt’s lymphoma (BL) [5] and acute lymphoblastic leukaemia (ALL), type B (B-cell ALL) and type T (T-cell ALL) [6]. This protocol emerged from a study which obtained promising results carried out in the MD Anderson Cancer Center, Houston [7, 8]. Subsequently its use became widespread internationally due to its encouraging therapeutic success. In the last few years, this general treatment applied to the three disorders has fallen into disuse in favour of a more specific therapy for each type of disease [9–11]. In particular, the protocol of the French group GELA [12] began to be used for MCL, the BURKIMAB regimen (2008, 2013 and 2014 versions) [13] for BL and the recommendations of the Spanish group PETHEMA (2003, 2008 and 2011 versions) for ALL, depending on age and cytogenetic risk [14, 15].

The synergistic effect of the various drugs used in these regimens exerts an anticancer action on malignant cancer cells, but also in healthy haematopoietic cells, which leads to considerable haematological toxicity in the form of anaemia, thrombocytopenia and neutropenia [16]. Therefore, a decrease in the three haematopoietic cell series (pancytopenia) can occur at an early stage, while patients are progressing to the period of post-chemotherapy aplasia (nadir). This can expose patients to a situation of severe neutropenia for several days or weeks; increasing the risk of contracting infections caused by different microorganisms, and causing a considerable increase in morbidity and mortality rates [17]. These drug combinations also have significant non-haematological toxicity (hepatic, haemorrhagic, neurological, gastrointestinal and mucocutaneous) [18].

The only study to have analysed the outcomes of the H-CVAD/MTX-AraC regimen compared to a specific protocol for ALL similar to one of the current three (GELA, BURKIMAB, PETHEMA) is a comparative study in 2018 related to H-CVAD/MTX-AraC and the old PETHEMA-93, in which no significant differences were found [19]. However, the most updated versions of PETHEMA in 2008 and 2011 have shown better results in terms of efficacy and safety compared to the traditional PETHEMA-93 version, so hypothetically they could be also better than H-CVAD/MTX-AraC [20]. Nonetheless, comparative studies between H-CVAD/MTX-AraC and the most modern versions of the PETHEMA 2008 and 2011 protocols, together with the other current regimens GELA and BURKIMAB, so far do not exist.

Taking into account these data, the aim of this study was to compare the safety results (haematological toxicity, non-haematological toxicity and infectious complications) for the only general protocol to those for the three recent specific regimens: H-CVAD/MTX-AraC versus GELA/BURKIMAB/PETHEMA, as well as the event free survival (EFS).

## Material and methods

### Study design

This was a retrospective and descriptive observational study comparing two groups of patients: those who were treated with H-CVAD/MTX-AraC (group A); and those who were treated with GELA/BURKIMAB/PETHEMA (group B). Data from group A patients were collected retrospectively, using the information compiled in their medical histories, while data from group B were collected prospectively.

### Patients

All patients aged from 18 to 69 years old who were diagnosed with MCL, BL, B-cell ALL or T-cell ALL treated and evaluated at Complejo Hospitalario Universitario de Albacete (CHUA) from 1 January 2002 to 31 December 2018 were included in the study.

On the one hand, group A patients received a single type of general chemotherapy treatment according to the H-CVAD/MTX-AraC regimen for all disorders, from 1 January 2002 to 31 December 2010. They were followed up throughout that period of time and afterwards. On the other hand, group B patients were studied from 1 January 2011 to 31 December 2018. They received a specific kind of protocol for each disease: French group GELA regimen for patients with MCL; BURKIMAB for those with BL and PETHEMA for those with B-cell ALL or T-cell ALL. Therefore, all patients were monitored for 5 years (median). In addition, the presence of disease progression or death was registered during the follow-up.

### Study variables and objectives

The following parameters were assessed in each study group:

- Haematological toxicity: anaemia, thrombocytopenia and neutropenia.
- Non-haematological toxicity: hepatic, haemorrhagic, neurological, gastrointestinal (diarrhoea) and mucocutaneous (mucositis).
- Infections and derived complications: number of patients affected by infection, infectious processes per group, episodes of febrile neutropenia, days of admission due to infection, delay of chemotherapy cycles due to infectious episode and death from sepsis/septic shock.
- EFS: 2-year and 5-year.

Based on these parameters, the main objective was to compare the EFS between the two groups. The secondary objectives were to compare the rate of infections and their complications, and the incidence of haematological or other toxicity, in both groups.

### Statistical analysis

To analyse the quantitative variables, the arithmetic mean was used as the measure of central tendency and the standard deviation as the measure of dispersion. For qualitative variables, the absolute and relative frequency as a percentage was used for each of the variables, which were reflected in frequency tables and bar charts.

The quantitative variables were compared in groups using Student’s *t* test. Contingency tables with Fisher’s exact test were used to compare qualitative variables (appearance or not of a certain event in each group). In both cases, the level of statistical significance considered was 5% (*p* <0.05).

Statistical calculations were performed with the SPSS 15.0 software package and Microsoft Excel 2010 for Windows.

### Ethical considerations

This was an analysis that compared the current therapeutic regimens with an historical cohort of patients retrospectively, so it has not modified current treatment guidelines. The rules of the Declaration of Helsinki were followed and the confidentiality of the data was guaranteed.

The project and the informed consent forms for this study were submitted to and approved by the CHUA Independent Ethics Committee.

## Results

### Composition of the analysed sample

The study included a total of 81 patients diagnosed with haematological malignancies: 42 (51.9%) were treated with H-CVAD/MTX-AraC from 2002 to 2010 (group A) and 39 (48.1%) were treated with the specific treatment regimens for each disorder from 2011 to 2018 (group B). Group A patients received a total of 259 treatment cycles, while group B patients received 194 cycles (Table 1).

In group A, 13 patients (31.0%) with MCL, 8 (19.0%) with BL, 14 (33.3%) with B-cell ALL and 7 (16.7%) with T-cell ALL were included. In group B, 9 patients (23.1%) with MCL, 10 (25.6%) with BL, 15 (38.5%) with B-cell ALL and 5 (12.8%) with T-cell ALL were included. There were not differences in the distribution of patients between the two groups (*p*-value = 0.770), so these are balanced out in respect to their composition by disorder (Table 2).

### Baseline characteristics at diagnosis

**Image 1.**
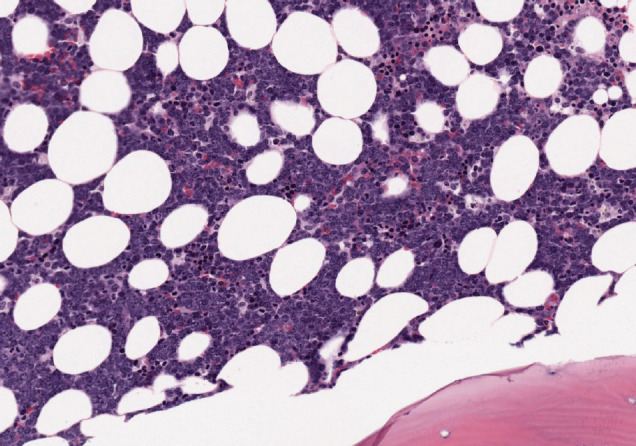
Bone marrow infiltration HE in MCL.

**Image 2.**
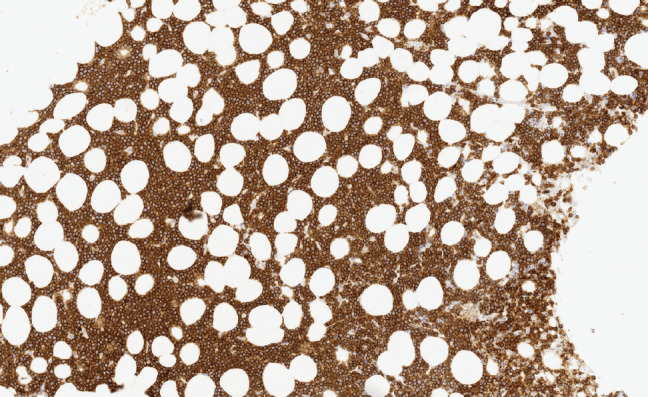
Bone marrow infiltration CD20(+) in MCL.

**Image 3.**
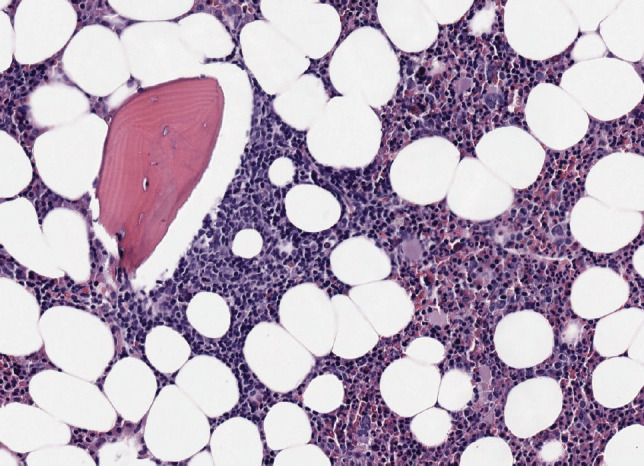
Bone marrow infiltration HE in BL.

**Image 4.**
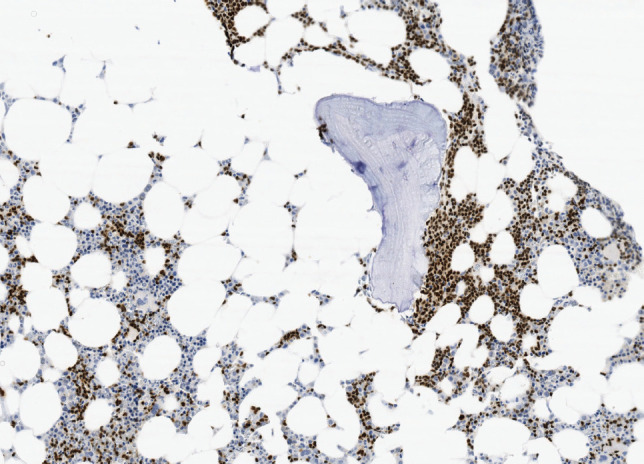
Bone marrow infiltration PAX-5(+) in BL.

- MCL (8 H-CVAD/MTX-AraC cycles versus 7 GELA cycles Þ AutoTPH):
  - Median age: 59 versus 56 years.
  - State of the disease: 100% lymphadenopathy (same distribution: laterocervical 61.5% versus 66.7%), bone marrow (BM) infiltration – stage IV – t(11;14) (84.6% versus 88.9%) (Images 1 and 2), splenomegaly (53.8% versus 44.4%), B symptoms (23.1% versus 22.2%).
- BL (8 H-CVAD/MTX-AraC cycles versus 4-6 BURKIMAB cycles):
  - Median age: 37 versus 38 years.
  - State of the disease: lymphadenopathy (37.5% versus 50% with the same distribution), ‘bulky’ mass (37.5% versus 30%), BM infiltration (50% versus 30%) (Images 3 and 4), stage IV (50% versus 50% due to infiltration of other organs), hypertransaminasemia due to hepatic infiltration (50% versus 40%), splenomegaly (25% versus 20%), B symptoms (37.5% versus 30%), human immunodeficiency virus (0% versus 30%).
- LAL-B (8 H-CVAD/MTX-AraC cycles versus 4 PETHEMA LAL-B cycles):
  - Median age: 36 versus 33 years.
  - State of the disease: 100% BM infiltration (obvious stage IV), B symptoms (78.6% versus 60%), high risk patients – complex karyotype (35.7% versus 60%).
- LAL-T (8 H-CVAD cycles versus 4 PETHEMA LAL-T cycles) :
  - Median age: 27 versus 26 years.
  - State of the disease: 100% BM infiltration (obvious stage IV), high risk patients – hyperleukocytosis and/or complex karyotype (71.5% versus 80%).

### Haematological toxicity

In group A, 100% of the patients had involvement of the three haematopoietic cell lines (anaemia, thrombocytopenia and neutropenia) (Table 3). Neutropenia was mostly severe (76.2% grade IV) and 23.8% grade III. Thrombocytopenia was also severe (47.6% grade IV and 52.4% grade III). In group B, the incidence of neutropenia was 74.4% (41.0% grade IV, 33.3% grade III). Half of patients (51.3%) had thrombocytopenia and 69.2% had anaemia. Differences between both groups regarding haematological toxicity were significant (*p* < 0.01) (Table 3).

### Non-haematological toxicity

Hepatic toxicity was higher in group B than group A (46.2% versus 21.4%, respectively, *p* = 0.033), and mostly grade II (72.2% of hepatic toxicities in 33.3% of all patients in group B) (Table 4). The incidence of haemorrhagic toxicity was significantly higher in group A (23.8% versus 5.1%, *p* = 0.027), with major bleeding 7.2% versus 0%, and minor bleeding 16.6% versus 5.1%. Neurological toxicity exclusively affected group A patients (14.3% versus 0%, *p* = 0.026). Conversely, diarrhoea only occurred in group B patients, although with low frequency (10.3% versus 0%, *p* = 0.049). Mucositis was very uncommon in the two groups (7.1% versus 5.1%, respectively, not significant) (Table 4).

### Infections

**Figure 1.**
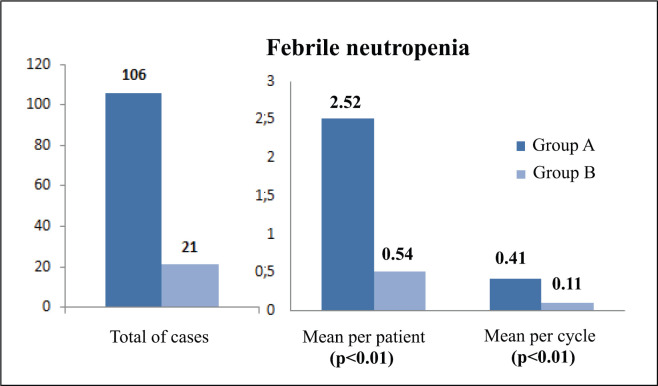
Comparison of febrile neutropenia between treatment groups.

The incidence of any infectious adverse event was similar in patients from both groups (81% versus 74.4%, respectively, *p* = 0.6) (Table 5). However, the total number of infections was higher in group A: 154 total events, 3.67 processes per patient and 0.59 episodes per cycle; compared to 48 events, 1.23 processes per patient and 0.25 episodes per cycle, in group B (*p* <0.01 for the three events). With regard to febrile neutropenia, in group A there were 106 cases, 2.52 per patient and 0.41 per cycle; while in group B, there were 21 cases, 0.54 per patient and 0.11 per cycle (*p* < 0.01) (Figure 1).

These infectious complications caused delay in the next chemotherapy cycle: 23 events, 0.55 per patient and 0.09 per cycle, in group A; compared to 11 events, 0.28 per patient and 0.06 per cycle, in group B (*p* <0.01); in addition to an average of 23 days of hospital admission per patient in group A versus 9 days in group B (*p* <0.01). Lastly, there were a total of five deaths caused by septic shock in group A patients versus one in group B (*p* = 0.2).

Bacteraemia was higher in group A: 30 (71.4%) versus 22 (56.4%) patients. In addition, the microbiological isolates in blood cultures were similar: Coagulase negative staphylococcus (38% versus 27%), *E. coli* (19% versus 20%), *E. coli* blee (10% versus 6.7%), *Klebsiella pneumoniae* (10% in group A) and *Campylobacter jejuni* (10% in group B).

### EFS : 2-year and 5-year

**Figure 2.**
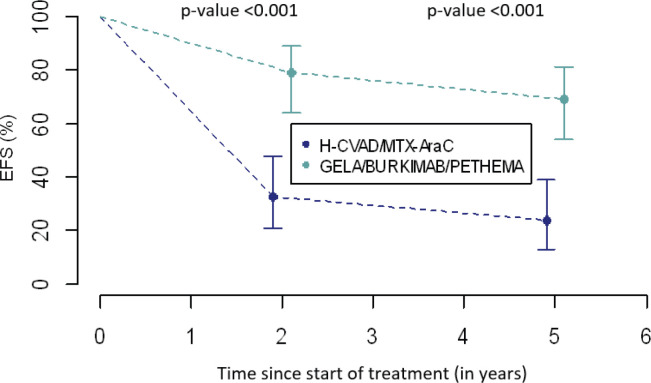
Comparison of EFS between treatment groups.

EFS 2-year and 5-year was higher in group B (Table 6 and Figure 2). In group A, 2-year EFS is 33% compared to 79% in group B (*p* <0.001). 5-year EFS is 24% versus 69%, respectively (*p* <0.001).

## Discussion

The treatment of haematological malignancies can lead to multiple complications due to its toxicity. Taking into account the aim of minimising its harmful effects, a number of progressively more specific therapeutic regimens have emerged for each type of disorder over the years [21]. The purpose of this study was, therefore, to analyse the differences in the safety profile between the different chemotherapy protocols, which yielded interesting results.

Firstly, haematological toxicity was significantly higher in patients treated with the general regimen (group A), occurring in all cases, and also in a more severe fashion. Due to the important implications in terms of infections, which will be discussed later, now we will focus on neutropenia [22]. All patients in this group developed neutropenia, most reaching grade IV. However, although most of the patients treated with the specific regimens (group B) also developed neutropenia, less than half of them had such a severe form. Approximately half of the patients in group B developed thrombocytopenia, but only a quarter of them grade IV, while in group A almost half of the patients manifested the most severe form. It was also found that a considerably smaller proportion of patients in group B developed anaemia compared to group A. Therefore, the specific therapeutic regimens showed less haematological toxicity and in less severe forms.

It was also interesting to compare non-haematological toxicity between the two groups. Hepatic toxicity was the most common form, especially in group B, occurring in almost half of the patients, although it was mild in most cases. This could be due to the administration of more hepatotoxic drugs, such as L-asparaginase [23], present in the current PETHEMA protocol but not in the H-CVAD/MTX-AraC one. To control this harmful effect, the corresponding drug dose adjustments are made according to the patient’s liver function, and it is monitored by changes in transaminase levels, without major incidents or clinical repercussions [24]. Another important aspect to highlight is haemorrhagic toxicity, which affected almost a quarter of group A. This alarming complication is probably favoured by the fact that around half of the group A patients had moderate/severe thrombocytopenia, leading to a risk of bleeding which can potentially be life-threatening, especially if it affects certain vital organs. It is also important to stress the complete lack of neurological toxicity of the specific regimens in our patients, compared to some cases in the general protocol group A which, although it was isolated, nevertheless it involved harmful complications and sequelae. In contrast, there was none related to gastrointestinal toxicity (diarrhoea) in group A patients, compared to isolated cases in group B, although almost all of them were self-limiting. The final aspect to mention is the anecdotal incidence of mucositis in both groups, without any clinical significance.

With this in mind, we would like to focus on the analysis of infections and their consequences which, as stated earlier, is the main objective of this study [25]. It is obvious that infectious complications continue to be the main cause of morbidity and mortality in these patients [26], as the vast majority manifested at least one episode during the course of their treatment, without differences between the two groups. However, this was the source of some of the most interesting questions we were able to explore. On the one hand, in group A there were three times more infectious processes in general, and per patient, compared to group B, with group B having half the number for each chemotherapy cycle versus group A. In addition, there were five times more cases of febrile neutropenia, in total and per patient, in group A (H-CVAD/MTX-AraC), compared to group B (specific regimens), with group B also having four-fold fewer cases per cycle. These data can probably be explained by the lower haematological toxicity found in the current protocols, especially in terms of neutropenia, which means that patients have a lower risk of contracting infections [27]. On the other hand, in group A, twice as many infectious events were the cause of delay in the next chemotherapy cycle compared to group B. This may be explained by a higher frequency and severity of febrile neutropenia that was previously found in group A, which leads to a slower and more difficult recovery from the infectious episode, complicating the ability to administer the next cycle. All the above factors may also have contributed to the higher number of days of hospital admission for infection required by patients in group A; in total almost three times the number, and per patient more than double, compared to group B. This means that these patients are hospitalised, away from their home environment, for longer periods of time, which may affect both their quality of life and their psychosocial well-being [28]. Moreover, these long hospital stays can increase the risk of contracting nosocomial infections and, in turn, could have economic consequences due to the increased occupation of hospital beds [29]. Finally, it was striking that no differences were found between groups in septic shock mortality rates, despite the fact that there were five times more deaths in patients treated with H-CVAD/MTX-AraC. However, one possible explanation for this apparent discrepancy could be the low number of deaths from septic shock in both cases, which makes the sample small in terms of achieving significant differences.

Finally, differences in 2-year and 5-year EFS between the two groups will be discussed. In group A, a third of the patients did not suffer from any event at 2 years into follow-up, in contrast, in group B more than three-quarters of patients did not exhibit any event. These results were corroborated at 5 years, a quarter of the former and almost three-quarters of the latter are free from any event, respectively. Seemingly, the combinations of drugs used in modern regimens have added some benefit to the treatment of these disorders specifically through their synergistic effect which was not present in the single regimen established for all chemotherapeutic cycles in general. Finally, new toxicity prevention strategies could play a very important role in this notable improvement of results [26, 27].

## Conclusion

In conclusion, lower haematological, neurological and haemorrhagic toxicity is found with the specific protocols. It is especially important to highlight the lower infection rate and milder profile with the current therapeutic regimens, which clearly benefit patients in many aspects of their daily life. In addition, they have longer EFS, which is an obvious long-term benefit. Therefore, this study has demonstrated a better safety profile of the newer regimens compared to H-CVAD and has now become the treatment of choice for many of our patients, confirming the great therapeutic advances in blood cancers in recent years [30]. Nevertheless, larger studies are necessary to confirm these results.

## Funding

The authors declare that they received no funding to carry out this study.

## Conflicts of interest

The authors declare that they have no conflict of interest.

## Figures and Tables

**Table 1.** Patients and cycles received according to disorder and treatment regimen.

Disorder	Total		Group A H-CVAD/MTX-AraC 2002–10		Group B specific regimens 2011–18	
LCM	22 patients	137 cycles	13 patients	75 cycles	9 patients	62 cycles
LB	18 patients	89 cycles	8 patients	42 cycles	10 patients	47 cycles
LAL-B	29 patients	143 cycles	14 patients	86 cycles	15 patients	57 cycles
LAL-T	12 patients	84 cycles	7 patients	56 cycles	5 patients	28 cycles
Total	81 patients	453 cycles	42 patients	259 cycles	39 patients	194 cycles

**Table 2.** Patients groups according to disorder.

Disorder	Group A		Group B		
	*N*	%	*N*	%	*p*-value
	42	100%	39	100%	0.770
LCM	13	31%	9	23.1%	
LB	8	19%	10	25.6%	
LAL-B	14	33.3%	15	35.8%	
LAL-T	7	16.7%	2	12.8%	

**Table 3.** Differences in haematological toxicity between the two treatment groups.

	Group A		Group B		
	*N*	%	*N*	%	*p*-value
Patients	42		39		
Neutropenia	42	100.0%	29	74.4%	**<0.001**
- Grade III	10	23.8%	13	33.3%	
- Grade IV	32	76.2%	16	41.0%	
Thrombocytopenia	42	100.0%	20	51.3%	**<0.001**
- Grade III	22	52.4%	10	25.6%	
- Grade IV	20	47.6%	10	25.6%	
Anaemia	42	100.0%	27	69.2%	**<0.001**

**Table 4.** Differences in non-haematological toxicity between the two treatment groups.

	Group A		Group B		
	*N*	%	*N*	%	*p*-value
Patients	42		39		
Hepatic toxicity	9	21.4%	18	46.2%	**0.033**
- Grade I	1	2.4%	1	2.6%	
- Grade II	6	14.3%	13	33.3%	
- Grade III	1	2.4%	3	7.7%	
- Grade IV	1	2.4%	1	2.6%	
Haemorrhagic toxicity	10	23.8%	2	5.1%	**0.027**
- Major bleeding	3	7.2%	2	0%	
- Minor bleeding	7	16.6%	0	5.1%	
Neurological toxicity	6	14.3%	0	0.0%	**0.026**
- Resting tremor Þ Vincristine suspension	1	2.4%	0	0.0%	
- Mild paresthesias (hands and feet)	3	7.2%	0	0.0%	
- Severe sensory motor neuropathy Þ Death	2	4.8%	0	0.0%	
Diarrhoea	0	0.0%	4	10.3%	**0.049**
Mucositis	3	7.1%	2	5.1%	0.999

**Table 5.** Mean number of infectious processes and associated characteristics.

Infections	Group A		Group B		
	*N*	%	*N*	%	*p*-value
Patients with some infectious process	34	81.0%	29	74.4%	0.595
Total of infectious process	154	100%	48	100%	**<0.01**
- Mean per patient	3.67		1.23		
- Mean per cycle of treatment	0.59		0.25		
Febrile neutropenia	106	68.8%	21	43.7%	**<0.01**
- Mean per patient	2.52		0.54		
- Mean per cycle of treatment	0.41		0.11		
Infectious events Þ delay CT cycle	23		11		**<0.01**
- Mean per patient	0.55		0.28		
- Media per cycle of treatment	0.09		0.06		
Total of days of admission due to infection	968	100%	352	100%	**<0.01**
- Mean per patient	23		9.0		
- Mean per cycle of treatment	3.7		1.8		
Death from septic shock	5	11.9%	1	2.6%	**0.2**

**Table 6.** EFS between treatment groups.

	Group A		Group B		
	Est.	95% CI	Est.	95% CI	p-value
EFS					
- At 2 years	33%	21%-48%	79%	64%-89%	**<0.001**
- At 5 years	24%	13%-39%	69%	54%-81%	**<0.001**

## References

[1] Sanz M, Carreras E, Rovira M (2019). Manual Práctico de Hematología Clínica.

[2] Chu E, Devita VT (2019). Physician´s cancer chemotherapy drug manual.

[3] Mathisen MS, Kantarjian HM, Jabbour EJ (2014). Emerging drugs for acute lymphocytic leukemia. Expert Opin Emerg Drugs.

[4] Romaguera JE, Fayad LE, Feng L (2010). Ten-year follow-up after intense chemoimmunotherapy with rituximab-HyperCVAD alternating with rituximab-high dose methotrexate/cytarabine (R-MA) and without stem cell transplantation in patients with untreated aggressive mantle cell lymphoma. Br J Haematol.

[5] Thomas DA, Kantarjian HM, Faderl S (2011). Hyper-CVAD and rituximab for de novo Burkitt lymphoma/leukemia. Blood.

[6] Thomas DA, O’Brien S, Faderl S (2010). Chemoimmunotherapy with a modified hyper-CVAD and rituximab regimen improves outcome in de novo Philadelphia chromosome-negative precursor B-lineage acute lymphoblastic leukemia. J Clin Oncol.

[7] Kantarjian HM, O’Brien S, Smith TL (2000). Results of treatment with hyper-CVAD, a dose-intensive regimen, in adult acute lymphocytic leukemia. J Clin Oncol.

[8] Fayad L, Thomas D, Romaguera J (2007). Update of the M. D. Anderson cancer center experience with hyper-CVAD and rituximab for the treatment of mantle cell and Burkitt-type lymphomas. Clin Lymphoma Myeloma.

[9] De Guibert S, Jaccard A, Bernard M (2006). Rituximab and DHAP followed by intensive therapy with autologous stem-cell transplantation as first-line therapy for mantle cell lymphoma. Haematologica.

[10] Oriol A, Ribera JM, Berqua J (2008). High-dose chemotherapy and immunotherapy in adult Burkitt lymphoma: comparison of results in human immunodeficiency virus-infected and noninfected patients. Cancer.

[11] Litzow MR (2011). Pharmacotherapeutic advances in the treatment of acute lymphoblastic leukaemia in adults. Drugs.

[12] Delarue R, Haioun C, Ribrag V (2013). CHOP and DHAP plus rituximab followed by autologous stem cell transplantation in mantle cell lymphoma: a phase 2 study from the Groupe d’Etude des Lymphomes de l’Adulte. Blood.

[13] Ribera JM, García O, Grande C (2013). Dose-intensive chemotherapy including rituximab in Burkitt’s leukemia or lymphoma regardless of human immunodeficiency virus infection status: final results of a phase 2 study (Burkimab). Cancer.

[14] Ribera JM, Oriol A, Morgades M (2014). Treatment of high-risk Philadelphia chromosome-negative acute lymphoblastic leukemia in adolescents and adults according to early cytologic response and minimal residual disease after consolidation assessed by flow cytometry: final results of the PETHEMA ALL-AR-03 trial. J Clin Oncol.

[15] Ribera JM, Morgades M, Ciudad J (2015). Post-remission treatment with chemotherapy or allogeneic hematopoietic stem cell transplantation (alloHSCT) of high-risk (HR) Philadelphia chromosome-negative (Ph-neg) adult acute lymphoblastic leukemia (ALL) according to minimal residual disease (MRD) preliminary results of the pethema ALL-HR-11 trial. Blood.

[16] Gill S, Lane SW, Crawford J (2008). Prolonged haematological toxicity from the hyper-CVAD regimen: manifestations, frequency, and natural history in a cohort of 125 consecutive patients. Ann Hematol.

[17] García Rodríguez JA, Gobernado M, Gomis M (2001). Guía clínica para la evaluación y el tratamiento del paciente neutropénico con fiebre. Rev Esp Quimoter.

[18] Díaz-Pedroche C, Salavert M, Aguado JM (2006). Evaluación individualizada del riesgo de infecciones en el paciente oncohematológico. Rev Esp Quimioter.

[19] Erkut N, Akidan O, Selim Batur D (2018). Comparison between Hyper-CVAD and PETHEMA ALL-93 in adult acute lymphoblastic leukemia: a single-center study. Chemotherapy.

[20] Ribera JM, Oriol A, Bethencourt C (2005). Comparison of intensive chemotherapy, allogeneic or autologous stem cell transplantation as post-remission treatment for adult patients with high-risk acute lymphoblastic leukemia. Results of the PETHEMA ALL-93 trial. Haematologica.

[21] Ribera JM, Montesinos P, Subirá M (2017). Pautas de quimioterapia en hemopatías malignas.

[22] Kuderer N, Dale DC, Crawford J (2007). Impact of primary prophylaxis with granulocyte colony stimulating factor on febrile neutropenia and mortality in adult cancer patients receiving chemotherapy: a systematic review. J Clin Oncol.

[23] Gokbuget N, Baumann A, Beck J (2010). PEG-asparaginase intensification in adult acute lymphoblastic leukemia (ALL): significant improvement of outcome with moderate increase of liver toxicity in the German Multicenter Study Group for Adult ALL (GMALL) Study 07/2003. Blood.

[24] Earl M (2009). Incidence and management of asparaginase–associated adverse events in patients with acute lymphoblastic leukemia. Clin Adv Hematol Oncol.

[25] Nesher L, Rolston KV (2014). The current spectrum of infection in cancer patients with chemotherapy related neutropenia. Infection.

[26] Crawford J, Dale DC, Lyman GH (2004). Chemotherapy induced neutropenia: risks, consequences, and new directions for its management. Cancer.

[27] De Naurois J, Novitzky-Basso I, Gill MJ (2010). Management of febrile neutropenia: ESMO clinical practice guidelines. Ann Oncol.

[28] Terol M, López S, Rodríguez J (2000). Diferencias en la calidad de vida: un estudio longitudinal de pacientes de cáncer recibiendo tratamiento de quimioterapia. Anales de Psicología.

[29] Kuderer NM, Dale DC, Crawford J (2006). Mortality, morbidity, and cost associated with febrile neutropenia in adult cancer patients. Cancer.

